# The Effect of Dust Storm on the Microbial Quality of Ambient Air in Sanandaj: A City Located in the West of Iran

**DOI:** 10.5539/gjhs.v7n7p114

**Published:** 2015-03-26

**Authors:** Heshmatollah Nourmoradi, Kambiz Moradnejadi, Fazel Mohammadi Moghadam, Behdad Khosravi, Lida Hemati, Ramin Khoshniyat, Farogh Kazembeigi

**Affiliations:** 1 Department of Environmental Health Engineering, School of Health, Ilam University of Medical Sciences, Ilam, Iran; 2 Rural Water and Wastewater Company. Ilam, Iran; 3 Department of Environmental Health Engineering, School of Health, Shahrekord University of Medical Sciences, Shahrekord, Iran; 4 Department of Environmental Health Engineering, Ilam University of Medical Sciences, Ilam, Iran; 5 Department of Environmental Health Engineering, School of Health, Kurdistan University of Medical Sciences, Kurdistan, Iran

**Keywords:** air pollution, dust storm, bacteria, fungi

## Abstract

**Background and Aims:**

The presence of pathogenic microorganisms in the dust storm can cause diseases such as Asthma, Pneumonia, and respiratory infections. The aim of this study was to determine the relationship between air-borne particles with airborne microorganisms in normal and dusty days in Sanandaj, a city located in the west of Iran.

**Materials and Methods:**

Air sampling was conducted during the normal and dusty days through Andersen single-stage impactor (28.3 L/min) for 2.5 min. Air particles concentration (PM_10_) was measured daily and microbial sampling was also conducted on every six days and on the dusty days. Finally, the data was analyzed by SPSS-16 (ANOVA and paired T-tests).

**Results:**

The concentration of airborne microorganisms (bacteria and fungi) was increased by an increase of the airborne particles. Particles concentration in May, June and July (twice per month) was more than of the standard value. The predominant species of bacteria and fungi during the occurrence of Dust storm was *Bacillus* spp. (56.2% of total bacteria) and *Mycosporium* spp. (28.6% of total fungi), respectively.

**Discussion and Conclusion:**

The results showed that the number of airborne microorganisms (bacteria and fungi) increased during the dust storm. Therefore, the microorganisms in the dust storm can cause biological harmful effects on human health.

## 1. Introduction

The North wind is the major cause of dust storms to Iran country, especially the west of Iran. This wind, which is active every year from May to September, is formed in the north of Middle East and after passing through the mountains of Turkey country and northern Iraq country, moves forward until it reaches to Iran (Shahsavani et al., 2012). Major natural sources of dust storm are tropical, subtropical and desert areas (Amarloei, Jonidi Jafari, Asilian Mahabadi, Asadollahi, & Nourmoradi, 2015). Among nine known regions in the world as the natural centers of dust storm, north of Africa is considered as the primary source which produces more than 50% of the airborne particles in the world. Tanaka and Chiba (2006) reported that the desert of Africa (Sahara desert) involves 58% of all the particles spreading on the Earth (Engelstaedter, Tegen, & Washington, 2006). Dust storm produced in the west of China and certain parts of Mongolia has been considered as the second biggest source of dust storm in the world (Ohara, Clarke, & Elatrash, 2006). The third source of dust storm causes by the Arabian Peninsula and its neighboring countries. This dust storm is the main cause of air pollutions in Iran (Goudie & Middleton, 2001). In the past, Iran, Iraq and Saudi Arabia countries jointly conducted mulching these regions at certain times of every year. But, in recent years, due to the various problems, mulching these regions has not been happened. Therefore, this phenomenon has caused the dust storms in Iran, especially the west of Iran (Goudie & Middleton, 2001). To the best of our knowledge, there were little studies conducted about Arabian dust storm. Draxler et al. (2001) reported that the average annual concentration of PM_10_ (particulate matter with aerodynamic diameter equal or less than 10 micrometer) in the air of Kuwait and Saudi Arabia countries, due to the Arabian dust storm, reached to 3000 µg/m^3^ (Draxler, Gillette, Kirkpatrick, & Heller, 2001). PM_10_ can cause adverse health effects including asthma, pneumonia and respiratory tract infections in human (Sandstrom & Forsberg, 2008). On the other hand, the microbial agents (bacteria and fungi) can travel long distances (5000 km) along with the airborne particles over the dust storms (Griffin, 2007; Prospero, Blades, Mathison, & Naidu, 2005; Bovallius, Roffey, & Henningson, 1980). Griffin and Kellogg (2006) showed that the microorganisms such as *Bacillus anthracis*, *Yersinia pestis*, *Mycobacterium tuberculosis*, *Legionella pneumophila* and influenza virus in the dust storms can cause harmful effects on human health (Kellogg & Griffin, 2006). In another study, Shahsavani et al. (2011) reported the dust storm increased the respiratory tract diseases and death rates in the dusty days of Ahwaz city, as one of the main cities of western Iran (Shahsavani et al., 2012). The aim of this study was to investigate the relation between air-borne particles with airborne microorganisms (bacterial and fungal organisms) in the normal and dusty days in Sanandaj city (Iran).

## 2. Materials and Methods

Sanandaj city is located in the western Iran (35°18′52″N, 46°59′32″E). The population of the city is 374,000 people. The sampling was conducted in a single-point at city center of Sanandaj during the first 6 months of 2012 from April to September at the height of 3 m from the land surface. Grim sampler (Dustcheck 1.108 portable dust monitor) was used to measure 24-hour average PM10 concentration. Anderson single-stage sampler (SKC Inc.) at air rate of 28.3 L/min for 2.5 min was used to determine bacteria and fungi in the air (Nourmoradi, Nikaeen, Amin, & Hatamzadeh, 2011). PM10 sampling was carried out daily but, the microbial sampling was taken every 7 days and also on the dusty days. Bacteria was cultured on heterotrophic plate count agar (HPA) at 37 °C for 48 hr. Sabouraud Dextrose agar (SDA) containing chloramphenicol antibiotic was also used to incubate fungal organisms at 25 °C for 3-5 days (Nourmoradi et al., 2011). Fungal organisms were distinguished microscopically (13). Completed culture media including Lowenstein-Jensen culture Medium for *Mycobacterium* spp., Mueller Hinton Agar for *Pseudomonas* spp. and blood agar for *Bacillus* spp. were also used to determine airborne bacterial species. The data was analyzed using SPSS-16 (ANOVA and paired T-tests). The p-value of 0.05 was considered as significant.

## 3. Results

**Figure 1:**
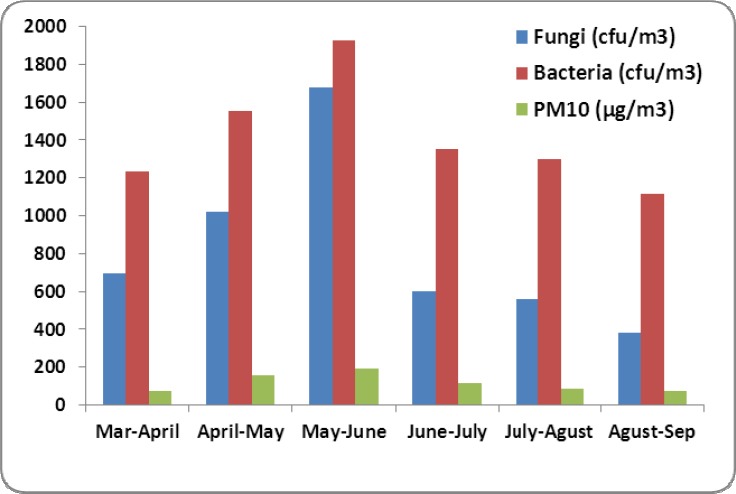
Airborne fungi, bacteria and PM10 concentration over the study

**Figure 2:**
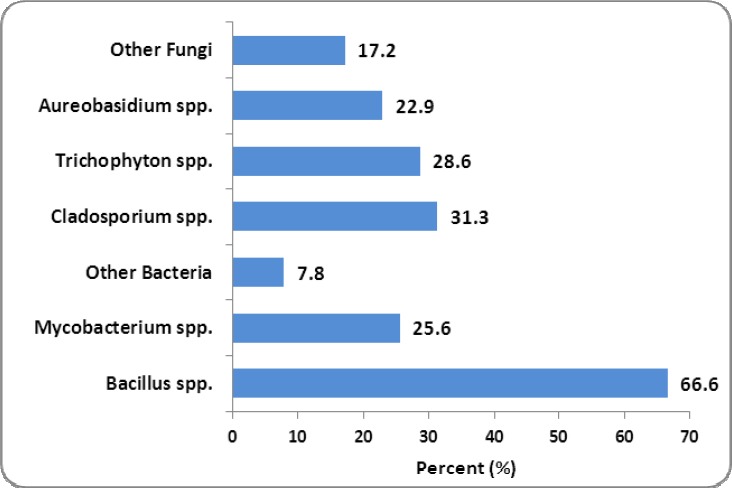
Airborne fungal and bacterial microorganisms in the normal days.

**Figure 3:**
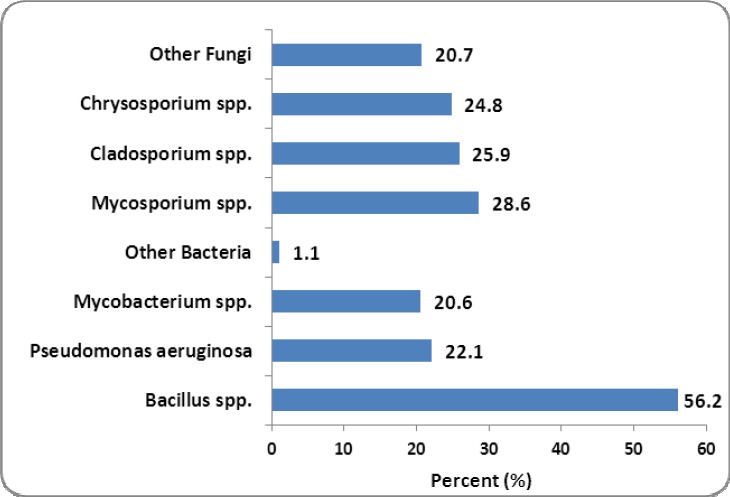
Airborne fungal and bacterial microorganisms in the dusty days

Table 1 shows the PM10 concentration (μg/m^3^) and meteorological data of air during study. As seen, the maximum and minimum of the PM10 concentration were occurred at May-Jun (191.7 μg/m^3^) and at Mar-Apr (73.3 μg/m^3^), respectively. Fig 1 shows the airborne fungi, bacteria and PM10 concentration during the study. As seen, the concentration of bacteria was more than of fungi. Bacteria and fungi concentration was in the range of 1117-1927 cfu/m^3^ and 384-1679 cfu/m^3^, respectively. The relation between PM10 with bacterial and fungal organisms and the average wind velocity in normal and dusty conditions is presented in Table 2. Based on the results, Table 2, the relation between PM10 and the number of bacteria and fungi in the normal and dusty days was significant (p-value<0.001), except for bacteria concentration in the normal days (p-value=0.961). Fig 2 and 3 show the predominant species of bacteria and fungi in the normal and dusty conditions, respectively. As can be seen, the predominantbacteria detected in the normal and dusty days were *Bacillus* spp. (56.2-66.6% of total bacteria). The most common fungi species found in the normal and dusty days were *Cladosporium* spp. (31.3% of total fungi) and *Mycosporium* spp. (28.6% of total fungi), respectively.

**Table 1:** QUOTE PM_10_ concentration (µg/m^3^) and meteorological conditions of air during the study

Time	PM_10_ Concentration (µg/m^3^)				Mean wind velocity (m/s)	Maximum wind velocity (m/s)	Mean temperature (°C)

	Min	Max	Mean	St. Dev			
Mar-Apr	85.6	120.5	73.3	134.20	1.9	14	11.8
Apr-May	68.3	264.0	156.4	372.18	2.2	11	15.1
May-Jun	90.2	326.4	191.7	180.06	2.7	12	22.4
Jun-Jul	80.6	125.3	115.4	143.35	2.5	9	27.0
Jul-Aug	68.8	110.8	88.3	96.21	2.3	10	22.7
Aug-Sept	63.0	90.9	75.0	65.97	1.8	9	23.5

**Table 2:** Relation between PM_10_ with microbial parameters and wind during the study

Condition	Pollutant (cfu/m^3^)	Parameter	Mean	P-value
Normal Days	PM_10_	Bacteria (cfu/m^3^)	1324	0.961
		Fungi(cfu/m^3^)	592	0.000
		Wind (m/s)	2.29	0.000
Dusty Days	PM_10_	Bacteria (cfu/m^3^)	1995	0.000
		Fungi(cfu/m^3^)	2268	0.000
		Wind (m/s)	2.10	0.000

## 4. Discussion and Conclusion

This study showed that many microorganisms including bacteria and fungi were found along with Arabic dust storms. So, the number of microorganisms was increased as the airborne particles were raised. Particles concentration in May, June and July (twice per month) was more than of the standard value. In these months, as presented in Figure 1, the number of bacteria and fungi was increased. Weir et al. (2000) reported that pathogenic microorganisms can be transferred by dust storms over the worldwide (Weir-Brush, Garrison, Smith, & Shinn, 2004). According to the study by Jeon et al. (2011) in South Korea, there was a significant positive correlations between PM_10_ and culturable bacterial population levels during the days affected by Asian dust events (ADE) (Jeon, H. J. Kim, Jung, J. H. Kim, M. Y. Kim, & Y. P. Kim, 2011). The results of present study, Table 2, showed that the mean number of bacteria and fungi of air in dusty days was 1.5 times and 3.83 times of normal days, respectively. Griffin et al. (2003) showed that airborne microorganisms originated from Africa Sahara desert can be transmitted to Atlantic Ocean. They reported that the number of airborne microorganisms (bacteria and fungi) in dusty days was nearly 5 times of normal daysIn their study, *Bacillus* spp. and *Cladosporium* spp. were the most species of bacteria and fungi, respectively (Griffin, Kellogg, Garrison, Lisle, Borden, & Shinn, 2003). The results of present study, Figures 2 and 3, also showed that *Bacillus* spp. (56.2-66.6%) and *Microsporum* spp. (28.6%) and *Cladosporium* spp. (31.3%) were the predominant species of bacterial and fungal microorganisms detected over the normal and dusty days, respectively.*Bacillus* spp. due to their endospore and *Microsporums* spp. due to the existence of fat layer around the cell wall could tolerate the unfavorable environmental conditions such as sun’s ultraviolet radiation and low temperature. Furthermore, because of the high concentration of dust particles, the penetration of sun’s rays including ultraviolet over dust storm events is significantly reduced. This phenomenon can also increase the survival of the microorganisms in the air of dusty days (Rothschild & Mancinelli, 2001). Maki et al. (2010) found that *Bacillus* spp., due to having endospore, was the main species of bacteria during dust storms in Japan (Maki, Susuki, Kobayashi, Kakikawa, Tobo, & Yamada, 2010). Hara and Zhang (2012) reported that airborne bacterial concentrations in dusty days were 10^6^ to 1.6 × 10^7^ CFU per cubic meter, which were 1 to 2 times higher than of in normal days. They also presented that 16 to 40% of total bacteria in dust were viable. They concluded that the Asian dust is one of the most important processes to disperse airborne bacteria in the worldwide atmosphere (Hara & Zhang, 2012). Fungal and bacterial organisms could be transmitted to far distance areas from the sources of dust production. Wind and turbulence of air are the important factors to spread of biological particles that cause to transfer fungi and bacteria from the original source to other areas (Lacey & West, 2007). In our study, there was no significant difference between the averagewind velocityduringdusty daysandnormal days (p-value>0.05). The present study showed that the mean velocity of wind was 1.8 to 2.7 meters per second. Kim and Chung (2010) reported that the wind velocity more than 8.0 meters per second results in the transfer of the dust particles from soil to the atmosphere (Kim & Chung, 2010). Shahsavani et al. (2000) found that the deserts of Iraq is the main source of Arabic dust storm in ahwaz city (Iran). They reported that during 72 days of the dust pollution in ahwaz city (Iran) in 2010, a total morbidity and mortality was 8157 and 1131 individuals, respectively (Shahsavani et al., 2012). Meng and Lu (2007) reported that there was a statistically significant relationship between dust storms and mortality due to cardiovascular and respiratory disease, respiratory hospitalization, upper respiratory tract infection, pneumonia, hypertension. Therefore, because of the presence of microorganisms along with PM_10_, it is proposed to determine the potential effects of dust storm on the human and also ecological health in this region (Sanandaj city, Iran).
